# Clinical efficacy of repetitive transcranial magnetic stimulation combined with electroacupuncture at Baliao points for functional constipation: a retrospective study

**DOI:** 10.3389/fneur.2026.1843563

**Published:** 2026-07-21

**Authors:** Lijie Gao, Yabei Shen, Shicai Huang, Xiangmei Gu, Mengjie Wang, Dong Fang, Sufen Han

**Affiliations:** Department of Colorectal Medicine, Kunshan Integrated Traditional Chinese and Western Medicine Hospital, Suzhou, Jiangsu, China

**Keywords:** Baliao points, brain-gut axis, electroacupuncture, functional constipation, neuromodulation, repetitive transcranial magnetic stimulation

## Abstract

**Background:**

Functional constipation (FC) is a prevalent gastrointestinal disorder with suboptimal treatment outcomes. Using a retrospective design, we examined whether adding repetitive transcranial magnetic stimulation (rTMS) to electroacupuncture (EA) at Baliao points was associated with greater clinical improvement than EA alone in patients with FC, and to generate hypotheses regarding combined neuromodulation.

**Methods:**

This retrospective cohort study included FC patients treated at a single hospital from February 2022 to March 2025, allocated by the treatment received to an observation group (rTMS combined with EA, *n* = 67) or a control group (EA alone, *n* = 75). The primary outcome was weekly complete spontaneous bowel movements (CSBMs); secondary outcomes were Patient Assessment of Constipation Symptoms (PAC-SYM), Patient Assessment of Constipation Quality of Life (PAC-QOL), Bristol Stool Form Scale (BSFS) and colonic transit time (CTT). Longitudinal outcomes were analyzed with linear mixed-effects models; confounding was addressed by propensity-score inverse-probability weighting, multivariable analysis of covariance and propensity-score matching; between-group effects are reported with 95% confidence intervals (CIs).

**Results:**

After 8 weeks, CSBMs increased more in the observation group (week-8 median 4.00 vs. 3.00; *p* < 0.001), as did improvements in PAC-SYM, PAC-QOL, BSFS and CTT (all *p* < 0.001). Mixed-effects models showed a significant group-by-time interaction for every repeated outcome (all *p* < 0.001). The differences persisted after adjustment for baseline covariates (adjusted week-8 CSBM difference 0.89, 95% CI 0.65 to 1.13; PAC-SYM − 6.18, 95% CI − 6.50 to −5.85). The proportion reaching ≥3 CSBMs/week did not differ between groups (92.5% vs. 88.0%, *p* = 0.37), whereas a large response (an increase of ≥3 CSBMs/week from baseline) was more frequent with combined treatment (67.2% vs. 17.3%; relative risk 3.87, 95% CI 2.30 to 6.53). Adverse events were mild and comparable between groups (22.39% vs. 18.67%, *p* = 0.583).

**Conclusion:**

In this retrospective, non-randomized cohort, adding rTMS to EA at Baliao points was associated with greater improvements in bowel frequency, symptom severity, quality of life and colonic transit, with comparable safety, and these associations were robust to propensity-score and multivariable adjustment. Given the absence of randomization, sham control and blinding, the findings should be interpreted as exploratory and hypothesis-generating and require confirmation in adequately powered, sham-controlled randomized trials.

## Introduction

Functional constipation (FC) is a common functional gastrointestinal disorder defined by the Rome IV criteria as a chronic condition characterized primarily by difficulty in defecation, reduced bowel movement frequency, and hard stool consistency in the absence of identifiable organic disease (1). Epidemiological surveys indicate that the global prevalence of FC is approximately 10–15%, with a substantial impact on patients’ quality of life and healthcare utilization (2). Despite the availability of multiple pharmacological and non-pharmacological treatment options, over half of adult patients report dissatisfaction with current therapies due to insufficient efficacy, adverse effects, and concerns regarding long-term medication dependence, underscoring the need for novel therapeutic strategies (3).

The pathophysiology of FC is multifactorial, involving abnormal colonic motility, visceral sensory dysfunction, and dysregulation of the brain-gut axis (4). Recent advances in neuromodulation have offered promising avenues for addressing these pathophysiological mechanisms. Repetitive transcranial magnetic stimulation (rTMS), a non-invasive neuromodulation technique, modulates cortical excitability through pulsed magnetic fields and has demonstrated potential in treating functional gastrointestinal disorders (5). rTMS targeting specific cortical regions, including the primary motor cortex (M1), has been shown to influence gastrointestinal motility through descending corticospinal pathways and modulation of autonomic nervous system activity (6). Specifically, high-frequency rTMS applied to the M1 hand representation area can enhance cortical excitability and promote vagal-mediated improvements in gastrointestinal function, providing a rationale for its application in FC (7).

**Figure 1:**
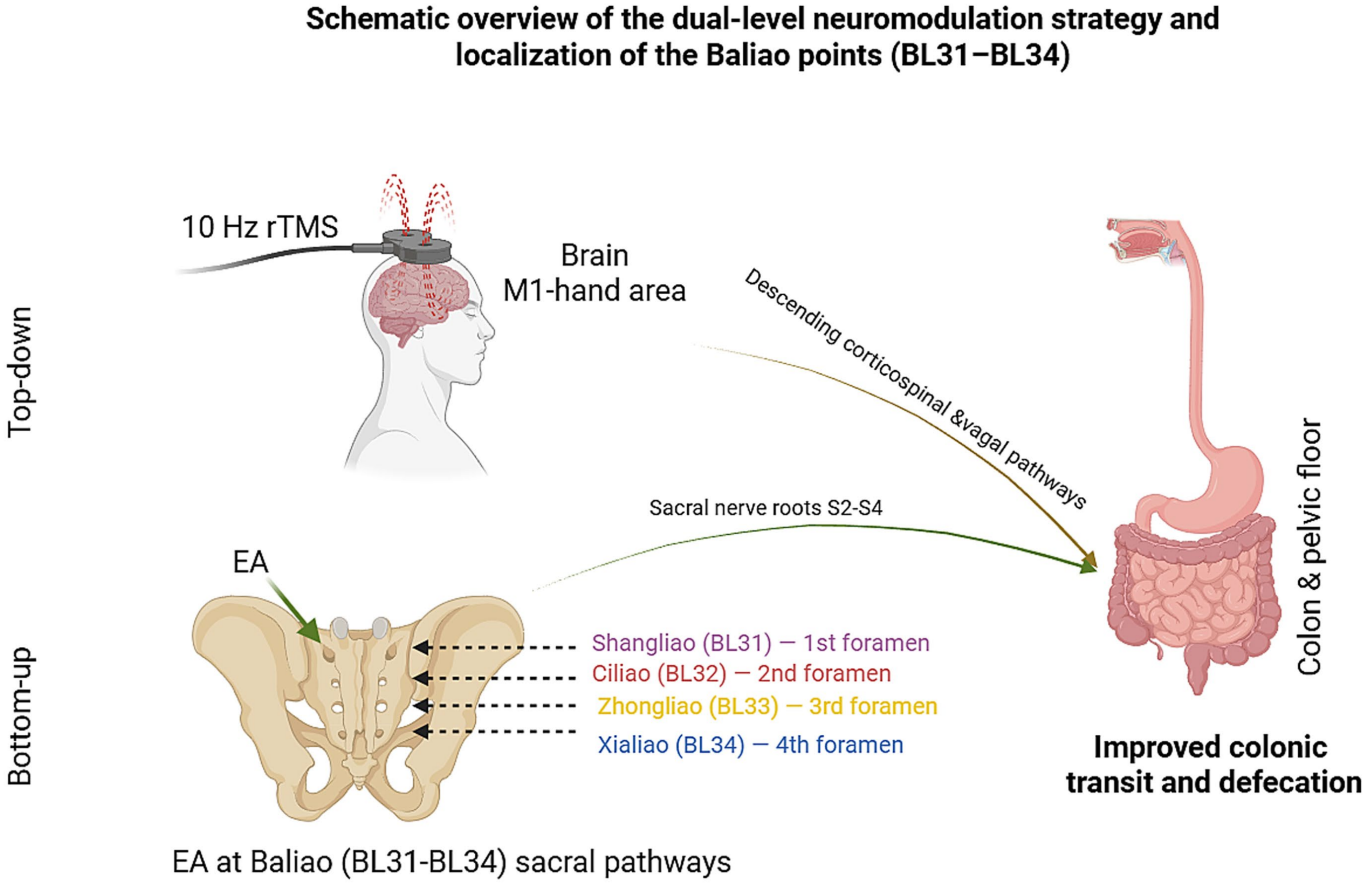
Schematic overview of the dual-level neuromodulation strategy and localization of the Baliao points (BL31–BL34). EA, electroacupuncture; M1, primary motor cortex; rTMS, repetitive transcranial magnetic stimulation.

Electroacupuncture (EA) represents another evidence-based neuromodulation approach for FC with established efficacy in clinical trials (8). The Baliao points, comprising four bilateral pairs of acupoints (Shangliao BL31, Ciliao BL32, Zhongliao BL33, and Xialiao BL34) located in the sacral foramina of the Bladder meridian, have traditionally been used for regulating pelvic organ function and treating defecatory disorders, as illustrated in Figure 1 (9). Modern research has demonstrated that EA at Baliao points directly stimulates sacral nerve roots, modulates anorectal dynamics, and improves pelvic floor coordination, thereby facilitating defecation through peripheral neuromodulatory pathways (10).

While individual studies have reported the efficacy of rTMS and EA for FC separately (11, 12), no investigation to date has evaluated the combined use of these two complementary neuromodulatory approaches. Theoretically, rTMS operates through central nervous system modulation (top-down regulation), whereas sacral EA exerts its effects predominantly via peripheral nerve stimulation (bottom-up regulation). This dual-level approach targeting both central and peripheral components of the brain-gut axis is hypothesized to yield additive or synergistic therapeutic benefits, although this hypothesis has not yet been tested in a randomized comparison. Therefore, this study aimed to retrospectively evaluate the clinical efficacy and safety of rTMS combined with EA at Baliao points for FC, providing preliminary evidence to inform the design of future prospective trials.

## Methods

### Study design and participants

This retrospective cohort study analyzed clinical data from FC patients treated at Kunshan Integrated Traditional Chinese and Western Medicine Hospital from February 2022 to March 2025. Patients who received rTMS combined with EA were assigned to the observation group, while those who received EA alone during the same period constituted the control group. Treatment assignment was determined by patient preference and physician recommendation at the time of initial consultation. The study protocol was approved by the Institutional Review Board (IRB No. KSYY2022-24). Due to the retrospective design, the requirement for informed consent was waived by the ethics committee.

#### Inclusion criteria

(1) Age 18–70 years; (2) FC diagnosed according to Rome IV criteria (13); (3) Symptom duration ≥ 6 months; (4) Baseline CSBMs < 3 per week; (5) Failure of at least one prior conventional treatment (dietary modification, laxatives, or biofeedback); (6) Complete medical records with documented 8-week follow-up data.

#### Exclusion criteria

(1) Secondary constipation attributable to medications (opioids, anticholinergics), metabolic disorders (hypothyroidism, diabetes), or neurological diseases (Parkinson’s disease, multiple sclerosis); (2) History of abdominal or pelvic surgery affecting gastrointestinal function; (3) Constipation-predominant irritable bowel syndrome (IBS-C) per Rome IV criteria; (4) Contraindications to rTMS, including history of seizures, intracranial metallic implants, cardiac pacemakers, or pregnancy; (5) Concurrent use of prokinetic agents, osmotic laxatives, or other neuromodulation therapies during the study period.

### Sample size considerations

As this was a retrospective study, formal prospective sample size calculation was not performed. However, with 67 and 75 patients in the observation and control groups respectively, a *post hoc* power analysis using a two-sided Mann–Whitney U test (*α* = 0.05) indicated that this sample provided approximately 85% power to detect a medium effect size (Cohen’s d = 0.5) between groups, which was considered adequate for this exploratory analysis.

### Treatment protocols

Both groups received conventional basic treatment comprising individualized dietary counseling emphasizing increased fiber and fluid intake, structured physical activity recommendations, and behavioral training for establishing regular bowel habits. Adherence to these dietary, fluid-intake, physical-activity and bowel-habit-training recommendations was advised and reinforced at each visit but was not systematically quantified (for example, by food-frequency questionnaire, food diary or activity monitor); the possibility of differential adherence between groups is therefore acknowledged as a limitation.

### Control group (EA alone)

Sacral EA therapy was performed targeting bilateral Shangliao (BL31, first sacral foramen), Ciliao (BL32, second sacral foramen), Zhongliao (BL33, third sacral foramen), and Xialiao (BL34, fourth sacral foramen) acupoints. With patients positioned prone, routine skin disinfection was performed, followed by insertion of sterile disposable acupuncture needles (0.30 mm × 50 mm, Hwato brand). Upon achieving de qi sensation, a Huatuo SDZ-V EA device was connected, delivering continuous wave stimulation at 2 Hz frequency at patient-tolerable intensity. Each session lasted 30 min and was administered once daily, 5 days per week during weeks 1–2, then 3 times per week during weeks 3–8. Needle insertion depths were standardized as follows: BL31, 25–40 mm; BL32, 50–60 mm; BL33, 40–50 mm; BL34, 25–30 mm, with the needle directed slightly medially and caudally to approximate the corresponding sacral foramen. The de qi sensation was operationally defined as the simultaneous occurrence of (i) a patient-reported sensation of soreness, distension, heaviness, or radiating warmth in the perineal or anorectal region, and (ii) a perception of needle grasp (‘needle heaviness’) by the operator. All EA procedures were performed by one of three licensed acupuncturists, each with at least 5 years of clinical experience in pelvic-floor acupuncture and trained in a unified Baliao-EA standard operating procedure before the study period in order to minimize operator-related variability.

### Observation group (rTMS + EA)

In addition to the identical EA protocol described above, rTMS was administered using a MagVenture MagPro X100 stimulator equipped with a figure-8 coil. The stimulation target was the left primary motor cortex hand representation area (M1-hand area), localized according to the international 10–20 electroencephalography system. The left M1-hand area was selected on three grounds: (i) the somatotopic representation of the hand provides a robust and reproducible cortical landmark that can be localized with surface anatomy and the 10–20 system, supporting reproducibility across operators; (ii) preclinical and clinical evidence indicates that high-frequency stimulation of M1 can modulate descending corticospinal and corticoautonomic pathways with downstream effects on gastrointestinal motility (6, 7); and (iii) M1 protocols carry an established safety profile in non-invasive neuromodulation. Alternative cortical targets that have been investigated in gut–brain axis research — including the dorsolateral prefrontal cortex (DLPFC) and the insular cortex — may also modulate visceral function through top-down emotional and interoceptive pathways and warrant direct comparison with M1 targeting in future prospective trials. Prior to the first treatment session, the resting motor threshold (RMT) was determined for each patient as the lowest stimulator output that produced motor-evoked potentials of at least 50 μV peak-to-peak amplitude in the contralateral first dorsal interosseous muscle in at least 5 of 10 consecutive single-pulse stimulations, recorded by surface electromyography. RMT was re-assessed before each subsequent rTMS session to account for day-to-day variability. Stimulation parameters were: frequency 10 Hz, intensity 100% of the individual RMT, train duration 5 s with 25-s inter-train intervals, 40 trains per session (2,000 total pulses), and total session duration of approximately 20 min. Treatment frequency matched the EA schedule. Each rTMS session was immediately followed by the EA protocol.

**Figure 2:**
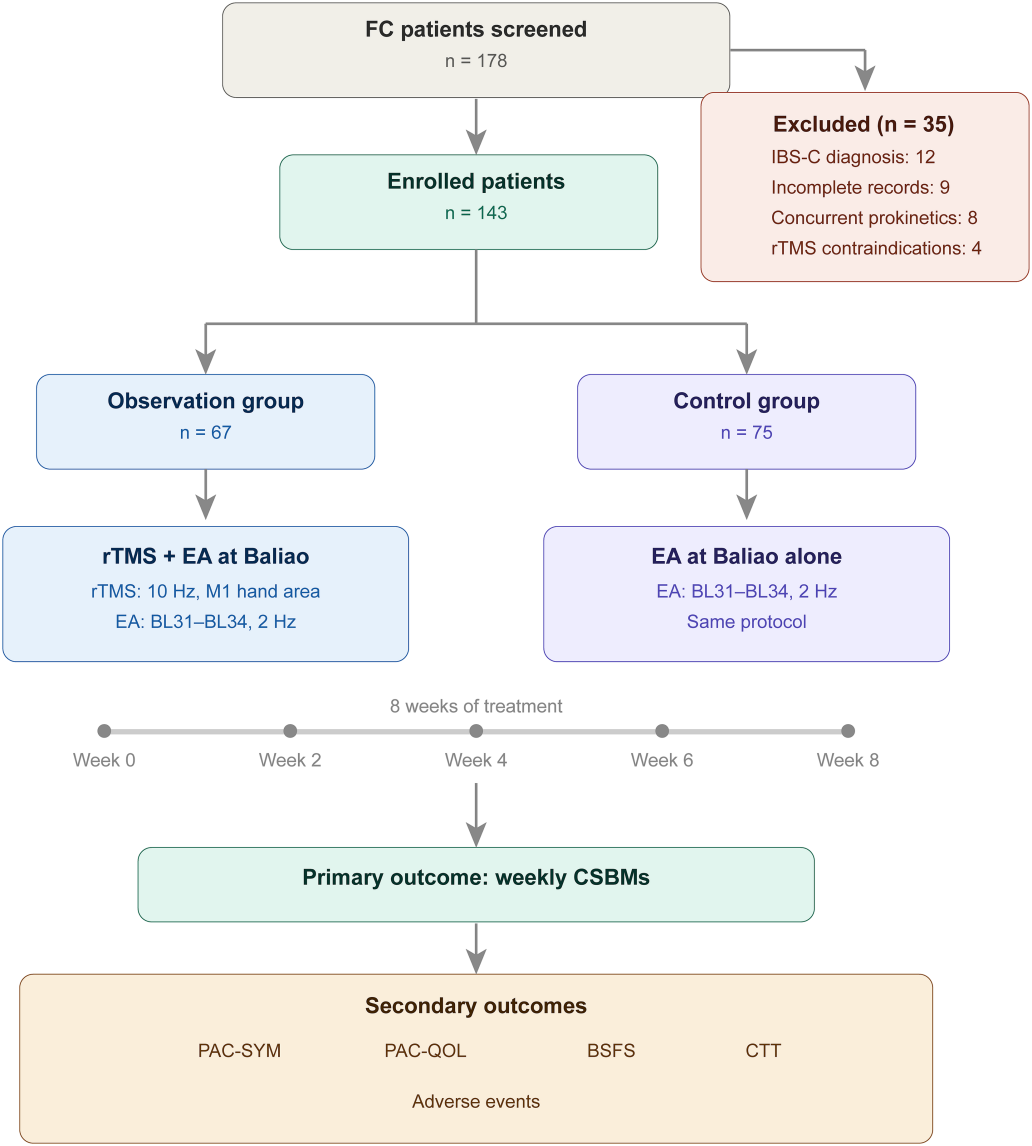
Study flowchart illustrating patient screening, group allocation (Observation vs. Control), intervention protocols, and outcome evaluation timeline. rTMS, repetitive transcranial magnetic stimulation; EA, electroacupuncture; CSBMs, complete spontaneous bowel movements.

The study flow and group allocation are illustrated in Figure 2.

## Outcome measures

### Primary outcome

The primary endpoint was the change in weekly CSBMs from baseline (pre-treatment) to week 8. CSBMs were defined as spontaneous bowel movements (without laxative use within the preceding 24 h) accompanied by a subjective sense of complete evacuation (14).

### Secondary outcomes

1. PAC-SYM assessed constipation symptom severity using 12 items across three domains (stool symptoms, rectal symptoms, abdominal symptoms) scored on a 5-point Likert scale (0 = absent, 4 = very severe), yielding total scores of 0–48, with higher scores indicating greater symptom severity (15).
2. PAC-QOL evaluated constipation-specific quality of life using 28 items across four domains (physical discomfort, psychosocial discomfort, worries/concerns, satisfaction) scored 0–4 (0 = not at all, 4 = extremely), yielding total scores of 0–112, with higher scores indicating more severe quality of life impairment (16).
3. BSFS assessed stool consistency on a 7-point visual scale (Types 1–2: hard/lumpy stools indicative of constipation; Types 3–5: normal consistency; Types 6–7: loose/liquid stools), scored 1–7 by type (17).
4. CTT was evaluated using the Metcalf radiopaque marker technique. Patients ingested standardized markers, and abdominal radiographs were obtained to calculate total transit time. Normal CTT is generally considered to be ≤72 h; values exceeding this threshold indicate delayed colonic transit (18).
5. Safety was assessed by systematic recording of all treatment-related adverse events throughout the 8-week study period. Events were retrospectively graded according to the National Cancer Institute Common Terminology Criteria for Adverse Events (CTCAE) version 5.0 (Grade 1, mild; Grade 2, moderate; Grade 3, severe; Grade 4, life-threatening; Grade 5, death). Each event was further classified by the most plausibly attributable modality: EA-related (e.g., needling pain, needle-site bruising, vasovagal symptoms), rTMS-related (e.g., scalp discomfort, transient headache or dizziness temporally linked to a stimulation session), or unrelated. Incidence was calculated as the proportion of patients experiencing at least one adverse event of any grade.

### Statistical analysis

Statistical analyses were performed using SPSS version 26.0 (IBM Corp, Armonk, NY, USA); the additional longitudinal, confounding-adjusted, responder and confidence-interval analyses added at revision were performed in Python 3.13 with the statsmodels (version 0.14) and SciPy libraries. Continuous variables were assessed for normality using the Shapiro–Wilk test. Normally distributed data were expressed as mean ± standard deviation (SD) and compared between groups using the independent samples *t*-test. Non-normally distributed data were expressed as median with interquartile range [M (Q1, Q3)] and compared using the Mann–Whitney U test, with effect sizes reported as rank-biserial correlations (*r*), where values of 0.1, 0.3, and 0.5 were considered small, medium, and large effects, respectively. Categorical variables were expressed as frequency (percentage) and compared using the χ^2^ test or Fisher’s exact test as appropriate. Because the primary and secondary endpoints were measured repeatedly over time (baseline and weeks 2, 4, 6 and 8 for CSBMs; baseline and weeks 4 and 8 for PAC-SYM and PAC-QOL), the longitudinal data were analyzed using linear mixed-effects models with a random intercept for each patient and fixed effects for treatment group, time (modelled as a categorical factor) and their interaction; the significance of the group-by-time interaction was evaluated with a likelihood-ratio test, and the model-based adjusted between-group difference at week 8 was obtained as a linear contrast with its 95% CI. To address potential confounding by indication arising from the non-randomized design, a propensity score for receiving combined treatment was estimated by logistic regression on age, sex, BMI, disease duration, prior laxative use and baseline anxiety/depression; covariate balance was summarized using standardized mean differences (SMD, with |SMD| < 0.1 considered well balanced) before and after weighting. The propensity score was then used in three complementary ways: stabilized IPTW, multivariable ANCOVA of the week-8 value adjusting for the corresponding baseline value and the above covariates, and 1:1 nearest-neighbor matching within a caliper of 0.2 SD of the logit of the propensity score as a sensitivity analysis. Between-group treatment effects are reported as adjusted mean differences (or, for the rank-based comparisons, Hodges-Lehmann median differences) together with 95% CIs. A responder analysis was performed, defining responders both as patients achieving ≥3 CSBMs/week at week 8 (symptom normalization) and, as a more discriminating threshold, as patients achieving an increase of ≥3 CSBMs/week from baseline (large response); groups were compared with the χ^2^ test and summarized using relative risk (RR), odds ratio (OR), risk difference and number needed to treat (NNT), each with 95% CIs. Given the multiple secondary outcome comparisons, the Benjamini-Hochberg procedure was applied to control the false discovery rate at 5%, and findings were interpreted with consideration of the overall pattern of results rather than individual *p* values in isolation. Statistical significance was set at two-sided *α* = 0.05. All reported *p* values are two-sided.

## Results

### Baseline characteristics

A total of 178 FC patients were screened, of whom 143 met the inclusion criteria and were included in the analysis (67 in the observation group and 76 in the control group; 1 patient in the control group was subsequently excluded due to incomplete follow-up data, yielding 75 analyzable patients). Reasons for exclusion included: IBS-C diagnosis (*n* = 12), incomplete medical records (*n* = 9), concurrent use of prokinetic medications (*n* = 8), rTMS contraindications (*n* = 4), and history of relevant surgery (*n* = 2). No significant differences in baseline demographics or clinical characteristics were observed between groups (all *p* > 0.05), supporting comparability (Table 1).

**Table 1:** Baseline characteristics comparison [*n* (%)/x̄±s].

Parameter	Observation group (*n* = 67)	Control group (*n* = 75)	t/Z/χ^2^	*p*-value
Age (years)	48.00 ± 8.00	47.17 ± 8.12	0.610	0.543
BMI (kg/m^2^)	24.92 (23.80, 26.29)	25.24 (24.00, 26.32)	0.468	0.640
Disease duration (months)	22.00 (16.00, 30.00)	20.80 (14.00, 30.60)	0.497	0.619
Gender, *n* (%)			0.043	0.835
Male	23 (34.33)	27 (36.00)		
Female	44 (65.67)	48 (64.00)		
Previous laxative use, *n* (%)			0.100	0.751
No	12 (17.91)	15 (20.00)		
Yes	55 (82.09)	60 (80.00)		
Anxiety/Depression, *n* (%)			1.853	0.173
No	42 (62.69)	55 (73.33)		
Yes	25 (37.31)	20 (26.67)		

### Changes in CSBMs

Table 2 presents CSBMs changes across the treatment period. Both groups demonstrated significant increases in weekly CSBMs from baseline (*p* < 0.05 at all time points within each group). The observation group showed significantly greater CSBM improvement compared with the control group beginning at week 4 (*p* < 0.001), and this difference persisted through weeks 6 and 8 (both *p* < 0.001). At week 8, the median CSBMs were 4.00 (3.00, 5.00) in the observation group versus 3.00 (3.00, 4.00) in the control group (*Z* = 3.677, *p* < 0.001, *r* = 0.37). The between-group difference at week 8 corresponded to a Hodges-Lehmann median difference of 1.0 CSBMs/week, and the difference in mean change from baseline was 0.88 CSBMs/week (95% CI 0.61 to 1.16).

**Table 2:** CSBMs changes (times/week).

Time point	Observation group (*n* = 67)	Control group (*n* = 75)	*Z*	*p*-value
Baseline (pre-treatment)	1.00 (1.00, 2.00)	1.00 (1.00, 2.00)	0.401	0.689
Week 2	2.00 (2.00, 3.00)ᵃ	2.00 (2.00, 2.00)ᵃ	1.557	0.120
Week 4	3.00 (2.00, 3.00)ᵃ	2.00 (2.00, 3.00)ᵃ	3.365	<0.001
Week 6	3.00 (3.00, 4.00)ᵃ	3.00 (3.00, 3.00)ᵃ	3.663	<0.001
Week 8	4.00 (3.00, 5.00)ᵃ	3.00 (3.00, 4.00)ᵃ	3.677	<0.001

𝐼ompared with baseline in the same group, *p* < 0.05 (Wilcoxon signed-rank test).

### Symptom assessment scale changes

Table 3 presents PAC-SYM and PAC-QOL score changes. Both scales demonstrated significant reductions from baseline in both groups. The observation group showed significantly greater improvements in PAC-SYM scores at both week 4 (*p* < 0.001, *r* = 0.47) and week 8 (*p* < 0.001, *r* = 0.75). Similarly, PAC-QOL improvements were significantly greater in the observation group at week 4 (*p* < 0.001, *r* = 0.47) and week 8 (*p* < 0.001, *r* = 0.68). All between-group comparisons remained significant after Benjamini-Hochberg correction for multiple testing. The between-group differences in mean change from baseline to week 8 were −6.07 points (95% CI − 6.42 to −5.73) for PAC-SYM and −15.02 points (95% CI − 15.69 to −14.35) for PAC-QOL.

**Table 3:** Symptom assessment scale score changes (points).

Scale	Time point	Observation group (*n* = 67)	Control group (*n* = 75)	*Z*	*p*-value
PAC-SYM	Baseline (pre-treatment)	25.00 (23.00, 26.50)	24.00 (23.00, 26.50)	0.601	0.548
	Week 4	19.00 (17.00, 20.00)ᵃ	21.00 (19.00, 22.00)ᵃ	5.684	<0.001
	Week 8	13.00 (11.00, 14.00)ᵃ	18.00 (17.00, 19.50)ᵃ	9.897	<0.001
PAC-QOL	Baseline (pre-treatment)	85.00 (79.50, 89.50)	83.00 (78.50, 89.50)	0.761	0.447
	Week 4	68.00 (64.50, 72.00)ᵃ	74.00 (70.00, 80.50)ᵃ	5.726	<0.001
	Week 8	56.00 (53.00, 60.00)ᵃ	68.00 (64.00, 73.50)ᵃ	9.033	<0.001

𝐼ompared with baseline in the same group, *p* < 0.05 (Wilcoxon signed-rank test).

### Stool consistency and colonic transit time changes

Table 4 presents BSFS and CTT outcomes. BSFS scores improved significantly more in the observation group (*p* < 0.001, *r* = 0.53), with median scores shifting from constipation-type (Type 2) to normal range (Type 4) compared with borderline improvement (Type 3) in the control group. CTT was significantly more reduced in the observation group (median 43.72 h vs. 55.02 h, *p* < 0.001, *r* = 0.61), with the observation group achieving a median CTT within the normal range (≤72 h) and demonstrating a substantially greater reduction from baseline. The between-group differences in mean change from baseline to week 8 were 1.16 points (95% CI 1.01 to 1.31) for BSFS and −15.50 h (95% CI − 16.73 to −14.26) for CTT.

**Table 4:** BSFS scores and CTT changes.

Parameter	Time point	Observation group (*n* = 67)	Control group (*n* = 75)	*Z*	*p*-value
BSFS score	Baseline (pre-treatment)	2.00 (1.00, 2.00)	2.00 (1.00, 2.00)	0.492	0.623
	Week 8	4.00 (3.00, 4.00)ᵃ	3.00 (2.00, 3.00)ᵃ	6.828	<0.001
CTT (hours)	Baseline (pre-treatment)	70.28 (63.81, 78.40)	68.76 (61.74, 78.63)	0.613	0.540
	Week 8	43.72 (39.69, 48.55)ᵃ	55.02 (49.54, 62.90)ᵃ	7.824	<0.001

𝐼ompared with baseline in the same group, *p* < 0.05 (Wilcoxon signed-rank test).

### Safety evaluation

The overall adverse event rate was 22.39% (15/67) in the observation group and 18.67% (14/75) in the control group, with no statistically significant between-group difference (χ^2^ = 0.302, *p* = 0.583). All recorded events were graded as CTCAE v5.0 Grade 1 (mild, self-limiting, not requiring intervention); no Grade ≥ 2 events occurred and no patient discontinued treatment because of an adverse reaction. When events were stratified by the most plausibly attributable modality (Table 5), rTMS-related events (scalp discomfort, transient headache or dizziness temporally linked to a stimulation session) occurred in 7/67 (10.45%) observation-group patients and, by definition, in none of the control-group patients; EA-related events (needling pain, needle-site bruising) occurred in 8/67 (11.94%) observation-group and 10/75 (13.33%) control-group patients. Two control-group patients reported scalp discomfort and two reported headache during the study period; in the absence of any cranial intervention these events were judged unlikely to be modality-related and are reported under the ‘EA / unrelated’ attribution column for transparency.

**Table 5:** Adverse event occurrence by CTCAE v5.0 grade and modality attribution [*n* (%)].

Adverse event (CTCAE v5.0 grade; attribution)	Observation group (*n* = 67)	Control group (*n* = 75)	χ^2^	*p*-value
Scalp discomfort — Grade 1; rTMS-related (obs)/unrelated (ctrl)	3 (4.48%)	2 (2.67%)		
Transient headache — Grade 1; rTMS-related (obs)/unrelated (ctrl)	3 (4.48%)	2 (2.67%)		
Transient dizziness — Grade 1; rTMS-related	1 (1.49%)	0 (0.00%)		
Mild pain during needling — Grade 1; EA-related	3 (4.48%)	5 (6.67%)		
Needle-site bruising — Grade 1; EA-related	5 (7.46%)	5 (6.67%)		
rTMS-related events, subtotal	7 (10.45%)	0 (0.00%)		
EA-related events, subtotal	8 (11.94%)	10 (13.33%)		
Patients with ≥ 1 adverse event (any attribution)	15 (22.39%)	14 (18.67%)	0.302	0.583

### Longitudinal mixed-effects analysis

In linear mixed-effects models with a random patient intercept, the group-by-time interaction was statistically significant for every repeated outcome, indicating that the between-group separation widened over the treatment course. For CSBMs, the group-by-time interaction at week 8 was 0.88 (95% CI 0.68 to 1.08; likelihood-ratio test for the overall group-by-time interaction, *p* < 0.001), and the model-based adjusted between-group difference at week 8 was 0.84 CSBMs/week (95% CI 0.55 to 1.13). The corresponding week-8 interaction terms were −6.07 points (95% CI −6.37 to −5.77) for PAC-SYM and −15.02 points (95% CI − 15.67 to −14.37) for PAC-QOL (both likelihood-ratio *p* < 0.001). Because these models account for the repeated-measures structure and the within-patient correlation that the pairwise Mann–Whitney and Wilcoxon tests do not, they provide a more appropriate analysis of the longitudinal data and reproduced the direction and approximate magnitude of the primary comparisons.

### Confounding-adjusted analyses

Before weighting, the treatment groups were well balanced on all measured covariates except baseline anxiety/depression (|SMD| = 0.23); after stabilized IPTW all covariates were balanced (all |SMD| < 0.1), and the propensity-score distributions overlapped. The adjusted between-group treatment effects at week 8 are summarized in Table 6. Multivariable ANCOVA, IPTW and 1:1 propensity-score matching (65 matched pairs) yielded effect estimates that were close to one another and to the unadjusted analyses for every outcome (for example, the adjusted CSBM difference was 0.89, 95% CI 0.65 to 1.13 by ANCOVA and 0.91, 95% CI 0.67 to 1.14 by IPTW), indicating that the observed associations were not explained by imbalance in the measured baseline covariates. These adjustments can balance only measured covariates and cannot account for unmeasured confounders, as discussed below.

**Table 6:** Confounding-adjusted between-group treatment effects at week 8 (observation vs. control).

Outcome	ANCOVA adjusted difference (95% CI)	IPTW adjusted difference (95% CI)	PS-matched difference (95% CI)	*P* (ANCOVA)
CSBMs (per week)	0.89 (0.65 to 1.13)	0.91 (0.67 to 1.14)	0.82 (0.38 to 1.26)	<0.001
PAC-SYM (points)	−6.18 (−6.50 to −5.85)	−6.17 (−6.48 to −5.87)	−6.40 (−7.24 to −5.56)	<0.001
PAC-QOL (points)	−14.82 (−15.46 to −14.18)	−14.88 (−15.48 to −14.29)	−14.02 (−16.40 to −11.63)	<0.001
BSFS (points)	1.20 (1.04 to 1.35)	1.18 (1.03 to 1.34)	1.09 (0.80 to 1.38)	<0.001
CTT (hours)	−15.41 (−16.45 to −14.36)	−15.36 (−16.38 to −14.34)	−14.36 (−17.62 to −11.10)	<0.001

ANCOVA, analysis of covariance adjusted for the baseline value, age, sex, BMI, disease duration, prior laxative use and baseline anxiety/depression. IPTW, stabilized inverse-probability-of-treatment weighting. PS, propensity score (1:1 nearest-neighbour matching within a 0.2-SD caliper; 65 matched pairs). Positive values favor the observation group for CSBMs and BSFS; negative values favor the observation group for PAC-SYM, PAC-QOL and CTT. All *p* values < 0.001.

### Responder analysis

Because both groups began with very low bowel frequency and improved over 8 weeks, the proportion reaching the normalization threshold of ≥3 CSBMs/week at week 8 was high and did not differ significantly between groups (observation 62/67, 92.5% vs. control 66/75, 88.0%; *p* = 0.37). When a more discriminating, magnitude-based definition was applied, however, a large response (an increase of ≥3 CSBMs/week from baseline) was achieved by 45/67 (67.2%) observation-group patients versus 13/75 (17.3%) control-group patients (RR 3.87, 95% CI 2.30 to 6.53; risk difference 0.50, 95% CI 0.36 to 0.64; NNT 2; *p* < 0.001). The additional benefit of combined treatment was therefore expressed chiefly as a greater magnitude of improvement rather than as a higher rate of simple symptom normalization.

## Discussion

This retrospective cohort study provides preliminary, hypothesis-generating evidence that the addition of rTMS to EA at Baliao points is associated with greater clinical improvement than EA alone in patients with FC. Across the primary outcome of weekly CSBMs and the secondary outcomes of symptom severity, quality of life, stool consistency, and colonic transit time, between-group differences favored the combined treatment, and the safety profile was comparable between groups. Because treatment was not randomized, a sham rTMS arm was absent, outcome assessment was not blinded, and propensity-score matching was not performed, the between-group differences reported here cannot be interpreted as a demonstration of superiority and should instead be regarded as a signal warranting confirmation in a prospective randomized trial. Reassuringly, the between-group differences were robust to propensity-score weighting, multivariable adjustment and propensity-score matching, making confounding by the measured baseline covariates an unlikely sole explanation; unmeasured confounding nonetheless remains possible.

The therapeutic rationale for this combined approach is grounded in the dual-level neuromodulatory concept targeting both central and peripheral components of the brain-gut axis. The brain-gut axis plays a fundamental role in the pathogenesis of FC, with dysregulation at multiple levels contributing to disordered colonic motility and impaired defecatory function (19). rTMS, by delivering high-frequency stimulation to the left M1-hand area, has been hypothesized to modulate cortical excitability and engage descending corticospinal and corticoautonomic pathways with potential downstream effects on gastrointestinal motility, although these pathways were not directly measured in the present study (6). This central, “top-down” modulation is complemented by the “bottom-up” effects of sacral EA, which is thought to stimulate sacral nerve roots (S2–S4) innervating the pelvic floor, rectum, and distal colon (10). By simultaneously targeting both the cortical regulatory centers and the peripheral sacral innervation, this combined approach may theoretically achieve more comprehensive modulation of the brain–gut axis than either modality alone, although the present design does not allow this mechanism to be directly tested. Accordingly, all references in this report to synergistic effects or to top-down/bottom-up brain–gut modulation are intended as mechanistic hypotheses to be tested in future studies incorporating autonomic, electrophysiological, neuroimaging or biomarker measurements, and not as conclusions supported by the present data, in which no mechanistic measurements were performed.

From the perspective of traditional Chinese medicine, FC falls within the diagnostic category of “constipation,” with pathogenesis involving large intestine dysfunction in conducting and conveying, related to factors including spleen-stomach weakness, qi stagnation, and yin-blood deficiency (20). The Baliao points, as Bladder meridian acupoints in the sacral region, serve functions of regulating lower jiao function and facilitating bowel movements (21). Modern anatomical studies have confirmed that needling at these points directly accesses the sacral nerve plexus, providing a mechanistic bridge between traditional meridian theory and contemporary neuroanatomy (10).

The observed time course of treatment response merits discussion. The between-group difference in CSBMs became statistically significant at week 4, suggesting that the additional benefit of combined treatment may require a cumulative treatment period to manifest. This pattern is compatible with — but does not establish — a proposed mechanism of neuroplastic adaptation, whereby repeated rTMS sessions are hypothesized to induce cumulative changes in cortical excitability and neural circuit function that may progressively enhance therapeutic effects (22); direct neurophysiological evidence supporting this mechanism in FC is currently lacking. The progressive widening of the treatment gap from week 4 through week 8 further supports this interpretation.

The magnitude of improvement across secondary outcomes was clinically meaningful. The PAC-SYM reduction in the observation group (median decrease of approximately 12 points) exceeded the established minimal clinically important difference (MCID) of 0.5 points per item or approximately 6 points total for this scale (15). Similarly, the shift in BSFS from Type 2 (hard, lumpy stools) to Type 4 (smooth, soft stools) represents a transition from constipation-pattern to normal stool consistency. The substantial CTT reduction (median decrease of approximately 26.5 h in the observation group vs. 13.7 h in the control group) provides objective physiological evidence of improved colonic motility beyond subjective symptom assessment.

The safety profile of the combined treatment was reassuring. The adverse event rates were comparable between groups, and all reported events were mild and transient. Notably, the rTMS-specific adverse events (dizziness and scalp discomfort) were consistent with those reported in prior rTMS studies and did not require treatment discontinuation (23). This favorable safety profile supports the feasibility of this combined approach for clinical application.

Several non-specific factors that could plausibly account for part of the observed between-group difference must be explicitly acknowledged. The observation group received approximately 20 additional minutes of therapeutic contact per session, including coil positioning, motor-threshold reassessment, and direct interaction with a clinician operating an unfamiliar device. Such additional attention, the novelty of an active neuromodulation device, and patient expectations are well-documented contributors to the placebo response in sham-controlled rTMS trials for functional gastrointestinal and pain conditions (24), and the present design cannot disentangle these effects from the specific neuromodulatory action of high-frequency M1 stimulation. The clinical magnitudes reported here should therefore be regarded as an upper bound on the genuine treatment-specific effect, and a sham-controlled randomized trial with blinded outcome assessment is required before any causal claim can be made.

## Limitations

Several important limitations of this study must be acknowledged. First, as a retrospective, non-randomized study, our findings are susceptible to selection bias and confounding. Treatment assignment was based on patient preference and physician recommendation rather than randomization, potentially introducing systematic differences between groups in unmeasured variables such as patient motivation, socioeconomic status, and disease severity beyond the parameters captured in baseline assessment. While baseline characteristics were comparable across measured variables, residual confounding from unmeasured factors cannot be excluded. To partially address confounding we undertook propensity-score-based adjustment (stabilized IPTW and 1:1 matching) and multivariable ANCOVA, after which the between-group differences were essentially unchanged. These techniques can, however, balance only measured covariates, and residual confounding from unmeasured factors—such as symptom severity beyond the recorded parameters, socioeconomic status, treatment expectations and health-seeking behavior—cannot be excluded.

Second, the relatively modest sample size (*n* = 142) limits statistical power for subgroup analyses and may not capture the full spectrum of treatment responses. Although our *post hoc* power analysis suggested adequate power for the primary comparison, the study was not powered to detect differences in adverse event rates or to identify patient subgroups that may preferentially benefit from combined treatment.

Third, the follow-up period was limited to 8 weeks, which precludes assessment of long-term treatment durability. Whether the observed benefits persist, diminish, or evolve following treatment cessation remains unknown and represents an important clinical question for future investigation.

Fourth, this study did not include a sham rTMS control group, making it impossible to distinguish the specific neurobiological effects of rTMS from non-specific effects such as placebo response, additional treatment time, and patient-provider interaction. The observation group received approximately 20 additional minutes of treatment contact per session, which may independently influence therapeutic outcomes through enhanced therapeutic relationship and expectation effects.

Fifth, while our outcome measures are validated and widely used in FC research, the study lacked objective neurophysiological assessments (e.g., anorectal manometry, electromyography, or functional neuroimaging) that could elucidate the mechanisms underlying the observed clinical improvements.

Finally, several variables that are known to influence symptom trajectories in functional constipation were not systematically captured in the medical records and could not be analyzed. Specifically: (i) adherence to dietary fiber and fluid recommendations was not quantified by food-frequency questionnaire or food diary, so differential compliance between groups cannot be excluded; (ii) habitual physical activity was not measured by an instrument such as the International Physical Activity Questionnaire (IPAQ); (iii) psychological comorbidity was captured only as a binary diagnosis at baseline without graded severity scoring such as the Hospital Anxiety and Depression Scale (HADS) or PHQ-9 / GAD-7, and within-treatment changes in mood — which can themselves modulate symptom reporting in brain–gut axis disorders — were not recorded; and (iv) use of rescue laxatives during follow-up was not prospectively logged with frequency and dose, so a differential rescue-medication burden cannot be ruled out as a contributor to the observed between-group differences. Each of these factors is plausibly associated with both treatment assignment and outcome and therefore represents a potential source of residual confounding. We have explicitly incorporated standardized capture of these variables into the protocol of the planned prospective randomized trial.

## Future directions

These preliminary findings provide a foundation for designing adequately powered, multicenter, prospective randomized controlled trials incorporating sham rTMS controls and blinding procedures. Future studies should systematically optimize the synergistic parameters of combined rTMS and sacral EA, including stimulation intensity, frequency, and treatment duration. Integration of functional magnetic resonance imaging and anorectal manometry would help elucidate the neurophysiological mechanisms underlying synergistic effects. Development of individualized treatment prediction models based on patient clinical profiles and neuroimaging biomarkers may ultimately facilitate precision medicine approaches to FC management.

## Conclusion

In this retrospective, non-randomized cohort study, the addition of rTMS to EA at Baliao points was associated with greater improvements in bowel frequency, constipation symptoms, quality of life, stool consistency, and colonic transit than EA alone, with a comparable safety profile. Because treatment was not randomized, no sham rTMS or blinded outcome assessment was used, and propensity-score matching or multivariable adjustment was not performed, these findings should be interpreted as exploratory and hypothesis-generating only. Confirmation in adequately powered, sham-controlled, prospective randomized trials with blinded assessment and standardized capture of dietary, activity, psychological, and rescue-medication confounders is required before clinical recommendations can be drawn. The associations were robust to propensity-score and multivariable adjustment for measured covariates but, given the non-randomized design, cannot establish causality.

## Data Availability

The original contributions presented in the study are included in the article/supplementary material, further inquiries can be directed to the corresponding author.
